# Hypoxia-induced miR-27 and miR-195 regulate ATP consumption, viability, and metabolism of rat cardiomyocytes by targeting PPARγ and FASN expression

**DOI:** 10.18632/aging.202778

**Published:** 2021-03-26

**Authors:** Jintuan Lin, Atikaimu Maimaitiyiming, Shaoxi Chen, Min Xiao, Zhanchao Xian

**Affiliations:** 1 ICU, Huazhong University of Science and Technology Union Shenzhen Hospital, Shenzhen, Guangdong, China; 2 ICU, People’s Hospital of Tajik Autonomous County, Tashkurgan, Xinjiang Uygur Autonomous Region, China; 3 Respiratory Department, The First People’s Hospital of Kashgar Region, Xinjiang Uygur Autonomous Region, China; 4 Department of Internal Medicine, People’s Hospital of Tajik Autonomous County, Tashkurgan, Xinjiang Uygur Autonomous Region, China; 5 Emergency Department, Shenzhen Hospital of University of Hong Kong, Shenzhen, Guangdong, China; 6 People’s Hospital of Tajik Autonomous County, Tashkurgan, Xinjiang Uygur Autonomous Region, China; 7 Department of Cardiology, Shenzhen Children’s Hospital, Shenzhen, Guangdong, China; 8 Department of Cardiovascular Medicine, Fuwai Hospital, Chinese Academy of Medical Science, Shenzhen, Guangdong, China

**Keywords:** hypoxia, cardiomyocytes, lipid metabolism, microRNA, ATP consumption

## Abstract

This study examined whether hypoxia-induced microRNA (miRNA) upregulation was related to the inhibition of chondriosome aliphatic acid oxidation in myocardial cells under anoxia. We showed that anoxia induced high expression of hypoxia-inducible factor-1-alpha, muscle carnitine palmitoyltransferase I, and vascular endothelial growth factor in cardiomyocytes. Meanwhile, miR-27 and miR-195 were also upregulated in hypoxia-induced cardiomyocytes. Furthermore, hypoxia induction led to reductions in the adenosine triphosphate (ATP) consumption rate and oxidative metabolism as well as an increase in cardiomyocyte glycolysis. Metabolic reprogramming was reduced by hypoxia, as evidenced by the downregulation of sirtuin 1, forkhead box protein O1, sterol regulatory element-binding protein 1c, ATP citrate lyase, acetyl-coenzyme A carboxylase 2, adiponutrin, adipose triglyceride lipase, and glucose transporter type 4, while miR-27 and miR-195 inhibition partially recovered the expression of these transcription factors. In addition, hypoxia induction reduced cell viability and survival by triggering apoptosis; however, miR-27 and miR-195 inhibition partially increased cell viability. Moreover, miR-27 and miR-195 targeted the 3’untranslated regions of two key lipid-associated metabolic players, peroxisome proliferator-activated receptor gamma and fatty acid synthase. In conclusion, miR-27 and miR-195 are related to hypoxia-mediated ATP levels, glycolysis, oxidation, cell survival, and a cascade of transcription factors that control metabolism in cardiomyocytes.

## INTRODUCTION

Obesity is one of the primary causes of heart issues and is associated with hyperlipidemia, hypertension, sleep-disordered breathing, and diabetes, all of which are key risk factors for coronary atherosclerosis [1]. However, a growing body of research suggests that some cardiovascular disorders are not directly caused by atherosclerosis but are associated with obesity [2]. Abnormal functioning of adipose tissue and heterotopic fat accumulation are the main factors that contribute to the progression of cardiovascular disease. The target genes of hypoxia-inducible factor (HIF) can contribute to lipid metabolism (LM) and are also involved in the pathogenesis of cardiovascular diseases [3]. The direct effect of hypoxia on cardiac function was experimentally studied in HIF-1α knockout mice. The results showed that HIF-1α could induce the activation of peroxisome proliferator-activated receptor gamma (PPARγ) as well as facilitate the development of metabolic reprogramming (MR) and systolic dysfunction under pathological conditions [4]. According to previous studies on human subjects and animal models, anoxia is related to the pulmonary vascular system, and pulmonary hypertension (PH) is partially caused by hypoxia [5]. Pulmonary vessel remodeling is associated with widespread MR, which influences the metabolism of saccharides and fat, ultimately leading to anoxia [6, 7]. In recent metabolomic studies, a mouse model of PH showed changes in lipid composition in the lungs through HIF-dependent MR [8].

MicroRNAs (miRNAs or miRs) are key regulatory factors in lipid formation, aliphatic acid oxidization, and lipoprotein synthesis and excretion. The maladjustment of miRNAs disrupts the gene regulation system, resulting in metabolic syndrome (MS) and related illnesses [9]. For instance, miR-30c reduces atheromatosis and hyperlipidemia by reducing lipoprotein excretion and lipid formation. The level of miR-30c evidently increases during lipogenesis and may play a pro-adipogenic role by upregulating the expression of lipocyte markers and enhancing lipid accumulation. The downregulation of miR-30c in ischemia and myocardial hypertrophy suggests that the expression of miR-30c may play an important role in determining the structure and normal functioning of the heart [10]. The expression of many genes coding for proteins involved in lipid metabolism, such as PPARα, fatty acid synthase (FASN), sterol regulatory element-binding protein 1 (SREBP-1), SREBP-2, apolipoprotein A1 (ApoA1), ApoB100, and ApoE3, was found to be inhibited by miR-27a. Hence, miR-27 may affect lipid metabolism by decreasing lipid formation and increasing lipid excretion from cells [11]. In the hearts of mice with streptozotocin (STZ)-induced type 1 diabetes, the expression of miR-195 was upregulated while that of miR-195-target proteins (sirtuin 1 [SIRT1] and B cell leukemia/lymphoma 2 [Bcl-2]) was downregulated. Inhibition of miR-195 reduced caspase-3 activity and oxidative pressure, improved myocardial function, and minimized myocardial hypertrophy in mice with STZ-induced diabetes with a concurrent increase in SIRT1 and Bcl-2 levels [12]. However, the miRNA-mediated relationship between cardiac hypertrophy and lipid biosynthesis remains unclear.

In this study, we investigated the effects of miRNAs on adenosine triphosphate (ATP) consumption, oxidative mitochondrial metabolism, glycolysis, lipid metabolism, and cell survival in hypoxia-induced disorders. Our results suggest that miR-27 and miR-195 are involved in regulating anoxia-induced cardiomyocyte lipid aggregation through the downregulation of PPARγ and FASN.

## RESULTS

### Hypoxia induced HIF-1α, mCPT-I, and VEGF expression in cardiomyocytes

**Figure 1:**
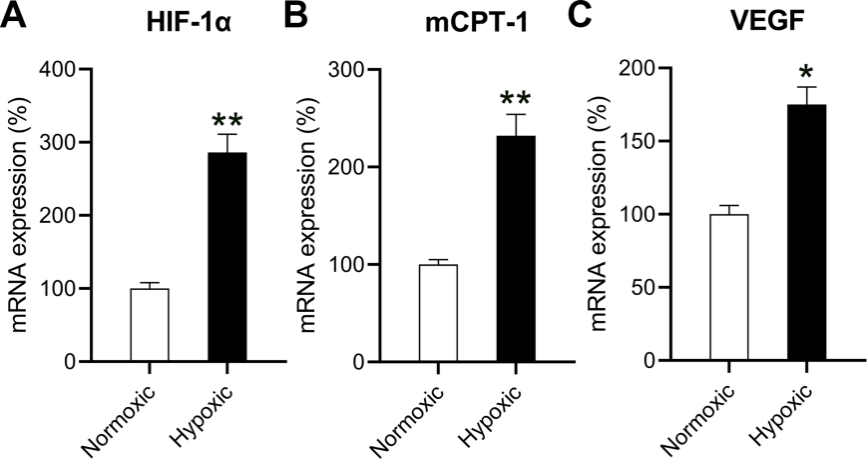
**Effects of hypoxia on the mRNA levels of HIF-1α, mCPT-I, and VEGF.** The isolated cardiomyocytes were maintained under normoxia and subjected to hypoxia for 48 h. The mRNA levels of (**A**) HIF-1α, (**B**) mCPT-I, and (**C**) VEGF were measured using RT-qPCR analysis (n = 3). *P < 0.05 and **P < 0.01 vs. the normoxia group.

Because hypoxia reduced the oxidation ability of mitochondrial fatty acids, leading to a decrease in chondriosome input and utilization of lipids—as well as build-up of neutral lipids in cardiomyocytes [10]—cardiomyocytes were independently exposed to hypoxic conditions (1% O_2_) and normoxic conditions (20% O_2_). The HIF-1α level in cardiomyocytes exposed to hypoxic conditions was significantly elevated compared to that in cardiomyocytes exposed to normoxic conditions, suggesting a successful induction (Figure 1A).

The rate-limiting steps of mitochondrial aliphatic acid input prior to funneling within the β-oxidation cycle are catalyzed by mCPT-I [13]. Therefore, we studied the influence of hypoxia on the expression of mCPT-I and found that hypoxia reduced mCPT-I mRNA levels (Figure 1B). VEGF is the target gene of HIF-1α [14], and anoxia induced VEGF expression (Figure 1C).

### Hypoxia induced miR-27 and miR-195 expression in cardiomyocytes

**Figure 2:**
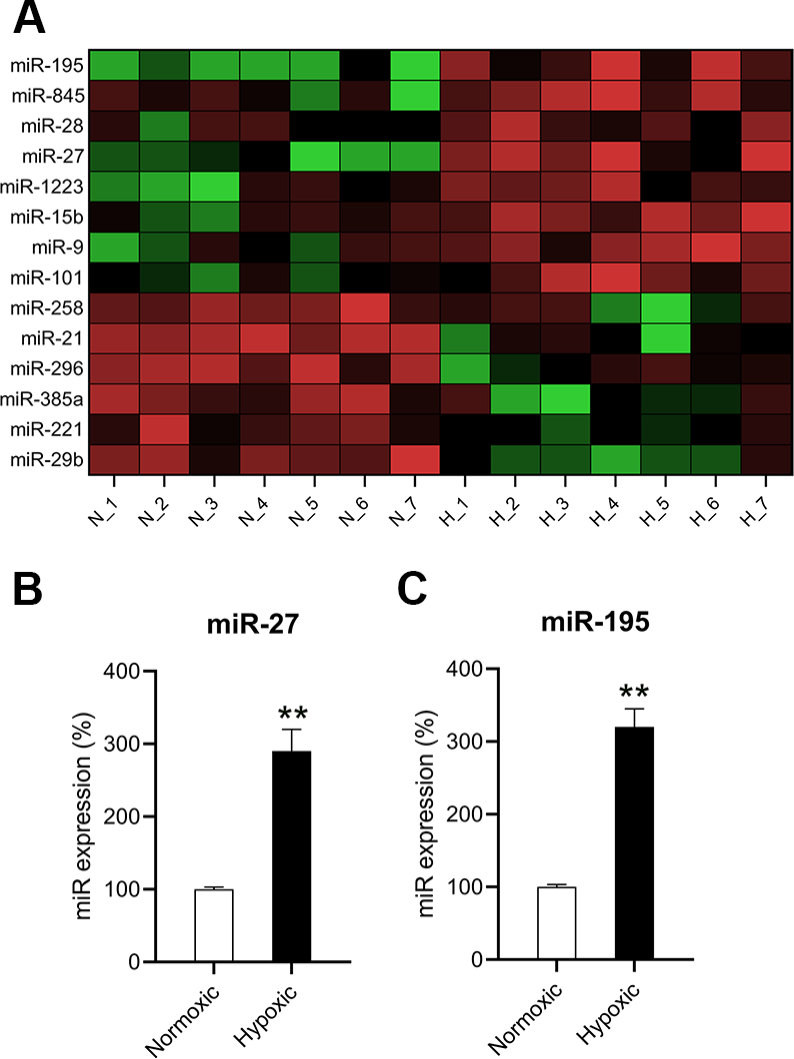
**miR-27 and miR-195 were upregulated in hypoxia-exposed cardiomyocytes.** (**A**) Microarray showed the differentially expressed genes in cardiomyocytes under normoxia (N; n = 7) and hypoxia (H,]; n = 7). (**B**, **C**) RT-qPCR was performed to show the expression of miR-27 and miR-195 in the isolated cardiomyocytes under normoxia and hypoxia (n = 3). **P < 0.01 vs. the normoxia group.

To understand the roles of miRNAs in hypoxia-induced cardiomyocytes, the differential expression levels of miRNAs were detected using microarray. The expression levels of several miRNAs were found to be different in hypoxia-induced cardiomyocytes compared to those in normoxia-conditioned cardiomyocytes. miR-27 and miR-195 were found to be upregulated (Figure 2A). RT-qPCR confirmed that miR-27 and miR-195 levels in hypoxia-induced cardiomyocytes were significantly upregulated compared to those in the normoxic group (Figure 2B, 2C).

### Inhibition of miR-27 and miR-195 promoted ATP consumption, glycolysis, and oxidation *in vitro*

**Figure 3:**
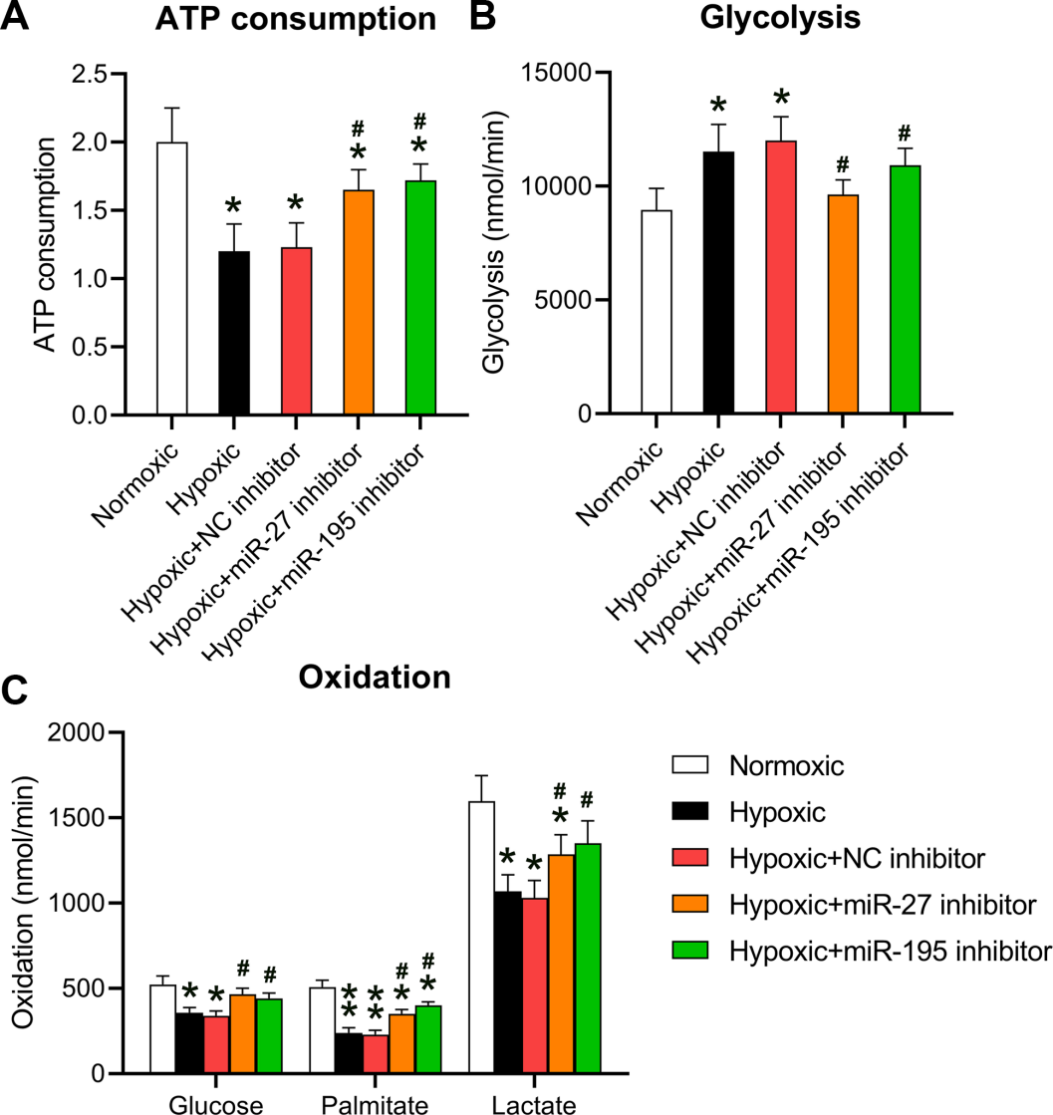
**Inhibition of miR-27 and miR-195 regulated hypoxia-affected mitochondrial metabolism and ATP consumption.** Isolated cardiomyocytes were transfected with NC, miR-27, and miR-195 inhibitors for 24 h followed by hypoxia treatment for 24 h. Cells under normoxia served as controls. (**A**) ATP consumption of cells in each group. (**B**) Glycolysis rates of the cells in each group. (**C**) Oxidation rates of glucose, palmitate, and lactate of the cells in each group (n = 3). *P < 0.05 vs. the normoxia group. ^#^P < 0.05 vs. the hypoxia group.

A previous study showed that hypoxia repressed cellular ATP consumption [15]. Here, the effect of miR-27 and miR-195 inhibition on the ATP consumption by cardiomyocytes was investigated. Cells grown under hypoxic conditions were transfected with miR-27 and miR-195 inhibitors. ATP consumption levels decreased in hypoxia-conditioned cells; however, both miR-27 and miR-195 inhibitors could partially restore ATP consumption (Figure 3A). Then, the role of miR-27 and miR-195 inhibition was investigated in mitochondrial metabolism. Hypoxic conditions were found to induce glycolysis compared to normoxic conditions. Additionally, inhibition of miR-27 and miR-195 resulted in decreased mitochondrial metabolism (Figure 3B). Consistent with the ATP consumption results, metabolic profiling of cardiomyocytes cultured under hypoxic conditions in the presence of miR-27 and miR-195 inhibition revealed prominent recovery in amylaceum, palmitate, and lactic acid oxidation (Figure 3C). The effect of miRNA mimics on normoxic conditions was also investigated; however, the mimics did not alter ATP consumption, glycolysis, and oxidation under normoxic conditions (data not shown). These data provide evidence that miR-27 and miR-195 are involved in hypoxia-mediated mitochondrial metabolism in cardiomyocytes.

### Inhibitory effect of miR-27 and miR-195 on cardiomyocyte MR

**Figure 4:**
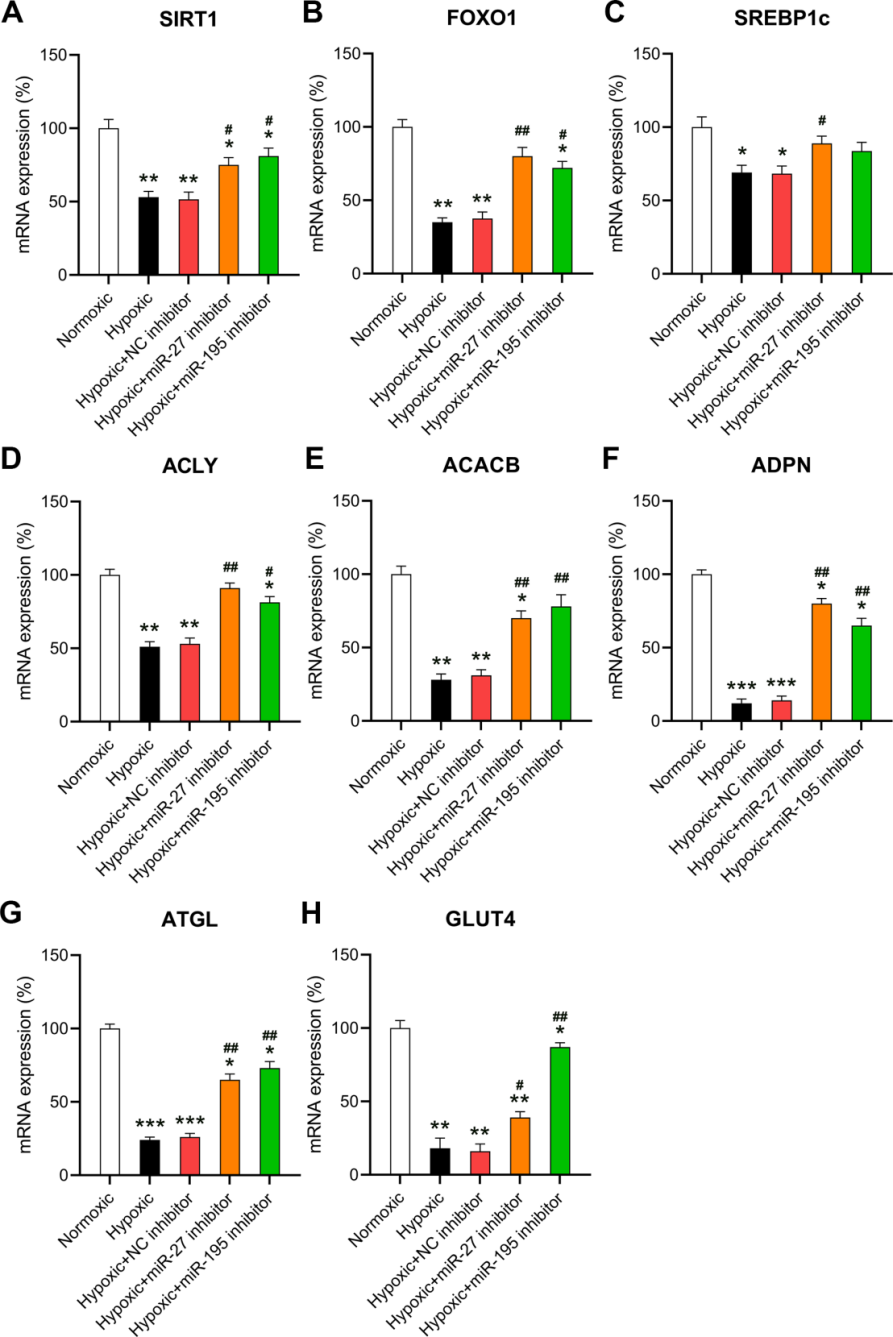
**Effect of miR-27 and miR-195 inhibition on hypoxia-influenced MR.** Isolated cardiomyocytes were transfected with NC, miR-27, and miR-195 inhibitors for 24 h followed by hypoxia treatment for 24 h. Cells under normoxia served as controls. RT-qPCR was performed to show the mRNA expression levels of (**A**) SIRT1, (**B**) FOXO1, (**C**) SREBP1c, (**D**) ACLY, (**E**) ACACB, (**F**) ADPN, (**G**) ATGL, and (**H**) GLUT4 in the isolated cardiomyocytes in each group (n = 3). *P < 0.05, **P < 0.01, and ***P < 0.001 vs. the normoxia group. ^#^P < 0.05 and ^##^P < 0.01 vs. the hypoxia group.

Given that miR-27 and miR-195 are involved in regulating hypoxia-induced changes in metabolism, changes in the key transcription pathways in response to miR-27 and miR-195 were also investigated. The core transcriptional pathways were tested in cardiomyocytes exposed to normoxic or hypoxic conditions in the presence or absence of miR-27 and miR-195 inhibition. A downregulation of two cardiac stress responses and metabolism coordinators [16, 17], i.e., SIRT1 and FOXO1 was observed in cells exposed to hypoxia (Figure 4A, 4B). In addition, inhibition of miR-27 and miR-195 partially rescued the expression of SIRT1 and FOXO1. In cardiomyocytes exposed to hypoxia, SREBP1c was significantly downregulated; however, SREBP1c expression was recovered upon inhibition of miR-27 and miR-195 (Figure 4C). The expression of ACACB and ACLY decreased significantly upon exposure to hypoxic conditions, but was restored by miR-27 and miR-195 inhibitors (Figure 4D, 4E). A similar trend was observed with genes encoding ADPN and ATGL (Figure 4F, 4G). ATGL is involved in lipid degradation, and mice lacking this gene exhibit fat accumulation and hypertrophic hearts [18]. Our findings indicate that the expression of GLUT4 and the phosphoenolpyruvate carboxykinase 1 decreased because of the lack of oxygen, but inhibition of miR-27 and miR-195 was able to reverse GLUT4 downregulation (Figure 4H). In conclusion, these results show that the LM of cardiomyocytes may be abrogated by hypoxia and restored by miR-27 and miR-195 inhibition.

### Effects of miR-27 and miR-195 inhibition on cardiomyocyte viability and apoptosis

**Figure 5:**
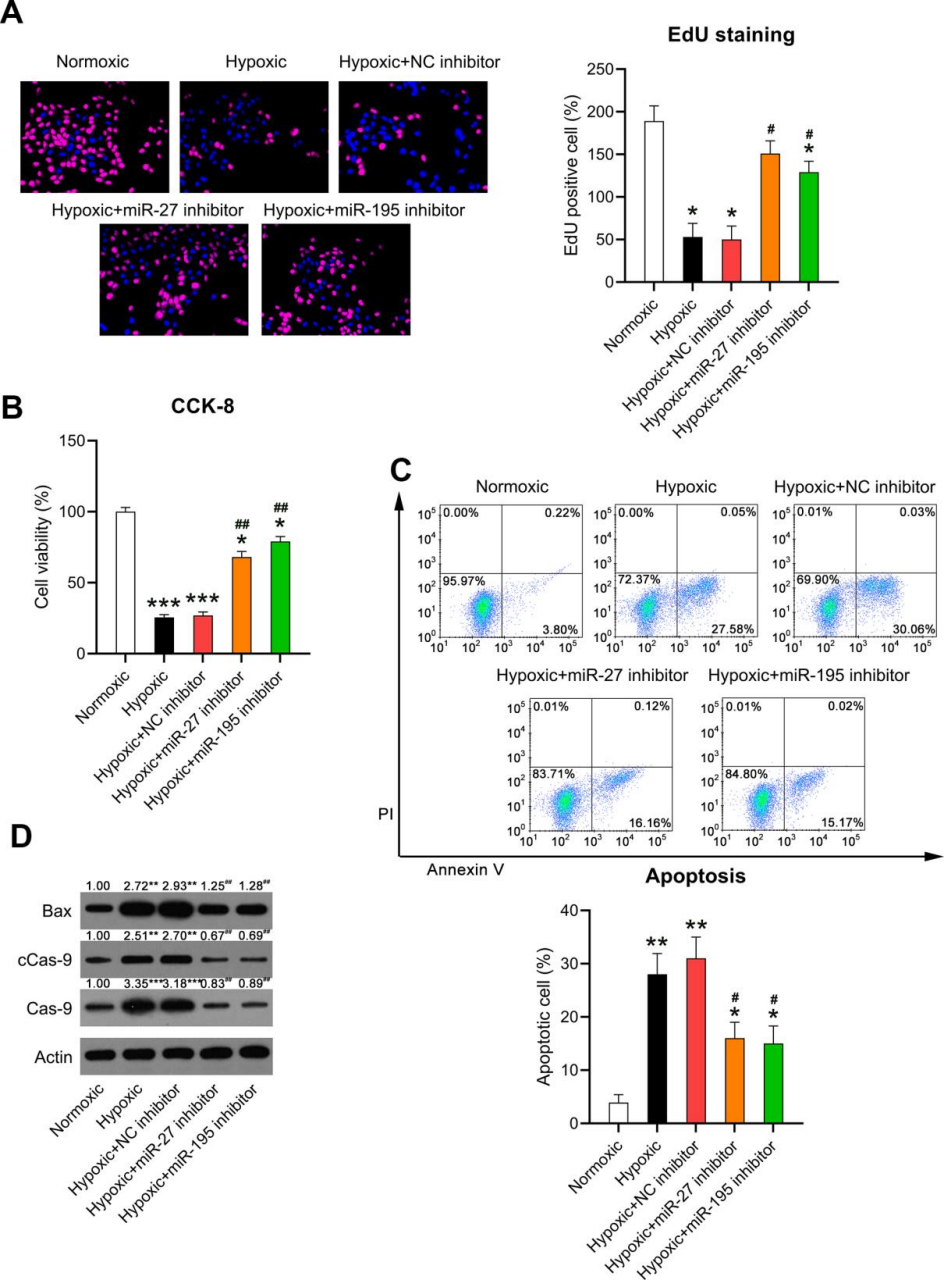
**Effect of miR-27 and miR-195 inhibition on the viability of hypoxia-induced cardiomyocytes.** Isolated cardiomyocytes were transfected with NC, miR-27, and miR-195 inhibitors for 24 h followed by hypoxia treatment for 24 h. Cells under normoxia served as controls. (**A**) EdU staining was performed to detect the proliferation of cells in each group. (**B**) The CCK-8 assay revealed the cell viability in each group. (**C**) Annexin V-FITC/PI flow cytometry was utilized to detect the apoptotic proportion of cardiomyocytes. (**D**) WB was utilized to detect the levels of Bax, caspase-9, and cleaved caspase-9 (n = 3). *P < 0.05, **P < 0.01, and ***P < 0.001 vs. the normoxia group. ^#^P < 0.05 and ^##^P < 0.01 vs. the hypoxia group.

Increased cardiomyocyte death is related to heart failure (HF) and can be related to apoptosis [19]. Given that cardiomyocyte survival can be suppressed by hypoxia, we evaluated whether miR-27 and miR-195 inhibition counteracted hypoxia-induced cell injury. EdU staining (Figure 5A) and the CCK-8 assay (Figure 5B) revealed that cell proliferation and viability were significantly reduced in cardiomyocytes exposed to hypoxia, respectively, while miR-27 and miR-195 inhibition partially restored the cell proliferation and viability of cardiomyocytes compared to those in cells transfected with the NC inhibitor.

It was hypothesized that miR-27 and miR-195 inhibition mediated hypoxia-induced cell death in cardiomyocytes. Annexin V-FITC/PI flow cytometry revealed increased apoptosis in hypoxia-exposed cardiomyocytes compared to that in the normoxia-exposed cardiomyocytes. However, inhibition of miR-27 and miR-195 remarkably reduced the apoptosis of hypoxia-exposed cells (Figure 5C). In addition, WB was performed to investigate caspase-9 cleavage and Bax expression. Hypoxia induced increased the cleavage of caspase-9 and Bax upregulation, while miR-27 and miR-195 inhibition decreased the cleavage of caspase-9 and downregulated Bax (Figure 5D), suggesting that miR-27 and miR-195 are involved in hypoxia-induced cardiomyocyte injury.

### miR-27 and miR-195 targeted the 3’-UTRs of PPARγ and FASN

**Figure 6:**
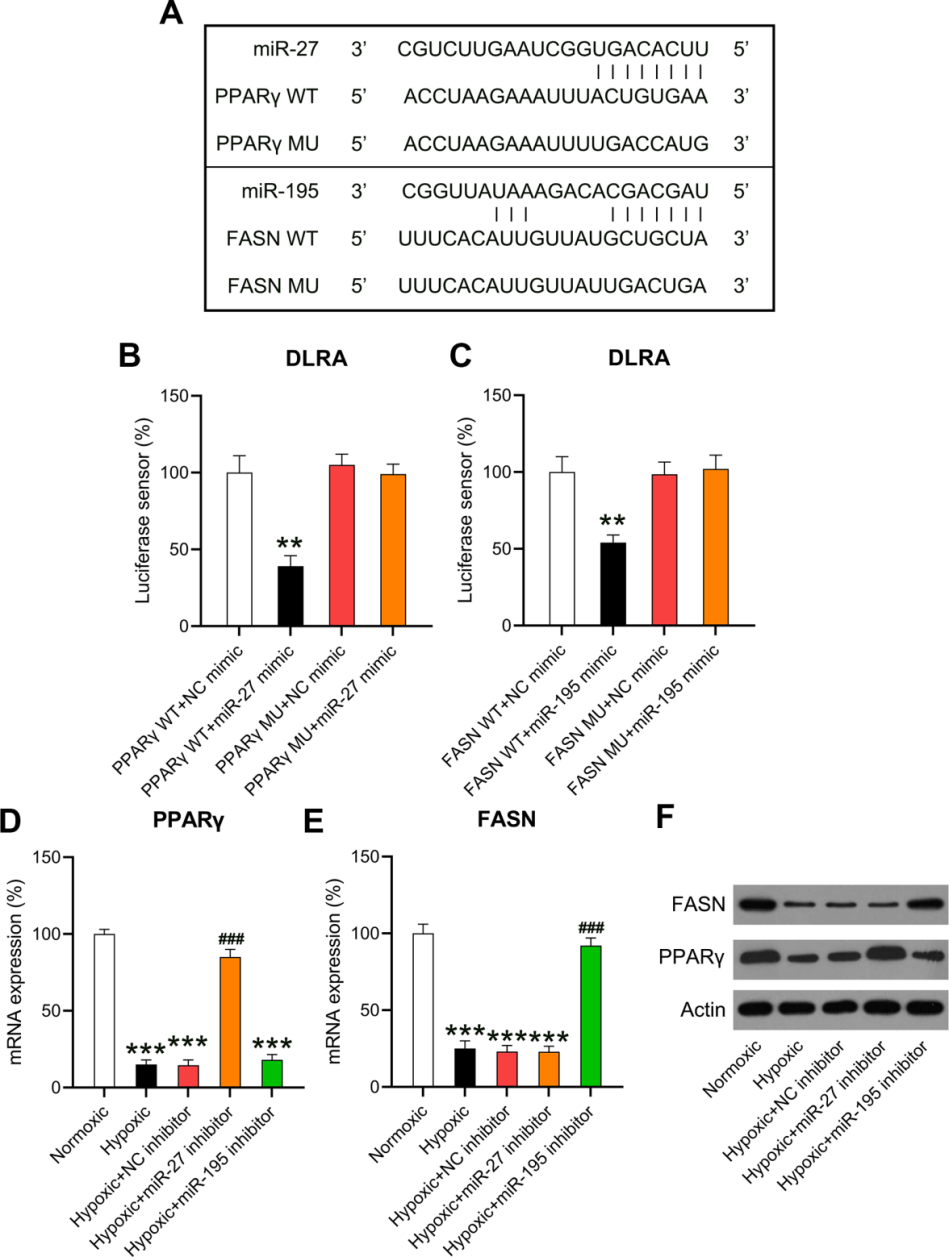
**miR-27 and miR-195 targeted the 3’-UTRs of PPARγ and FASN.** (**A**) Bioinformatic analysis showed that the 3’-UTRs of PPARγ and FASN had binding sites in the sequences of miR-27 and miR-195. (**B**, **C**) The DLRA was performed by co-transfection of luciferase reporter containing the WT or MU-binding sites of PPARγ and FASN with miR-27 and miR-195 mimics into cardiomyocytes. (**D**–**F**) Isolated cardiomyocytes were transfected with NC, miR-27, and miR-195 inhibitors for 24 h followed by hypoxia treatment for 24 h. Cells under normoxia served as controls. RT-qPCR and WB were utilized to detect the expression levels of PPARγ and FASN mRNA and protein in isolated cardiomyocytes in each group (n = 3). **P < 0.01 and ***P < 0.001 vs. the NC/normoxia group. ^###^P < 0.001 vs. the hypoxia group.

HF is attributed to changes in aliphatic acid metabolism and metabolic inflexibility, and aliphatic acid metabolism is closely associated with PPARγ and FASN [20]. Previous studies have suggested negative correlations between miR-27 and PPARγ [21] as well as miR-195 and FASN [22]. Bioinformatic analyses were performed to detect the miRNA targets. Both miR-27 and miR-195 targeted the 3’-UTRs of PPARγ and FASN (Figure 6A). DLRA revealed that co-transfection with miR-27 and miR-195 mimics inhibited luciferase activity by 55% and 45%, respectively—in luciferase constructs where the luciferase gene was downstream to the 3’-UTRs of WT PPARγ and FASN—compared to the NC simulation. Luciferase assay revealed that the binding regions of MU PPARγ and FASN did not show significant changes, indicating that miR-27 and miR-195 directly targeted PPARγ and FASN, respectively (Figure 6B, 6C). The expression of PPARγ and FASN in hypoxia-conditioned cardiomyocytes was investigated. RT-qPCR and WB revealed that PPARγ and FASN levels decreased remarkably in the hypoxia group compared to those in the normoxia group. However, the expression of PPARγ and FASN increased after miR-27 and miR-195 inhibition (Figure 6D–6F), suggesting that miR-27 and miR-195 targeted the 3’-UTRs of PPARγ and FASN.

### Effect of PPARγ and FASN overexpression on cardiomyocyte metabolism and death

**Figure 7:**
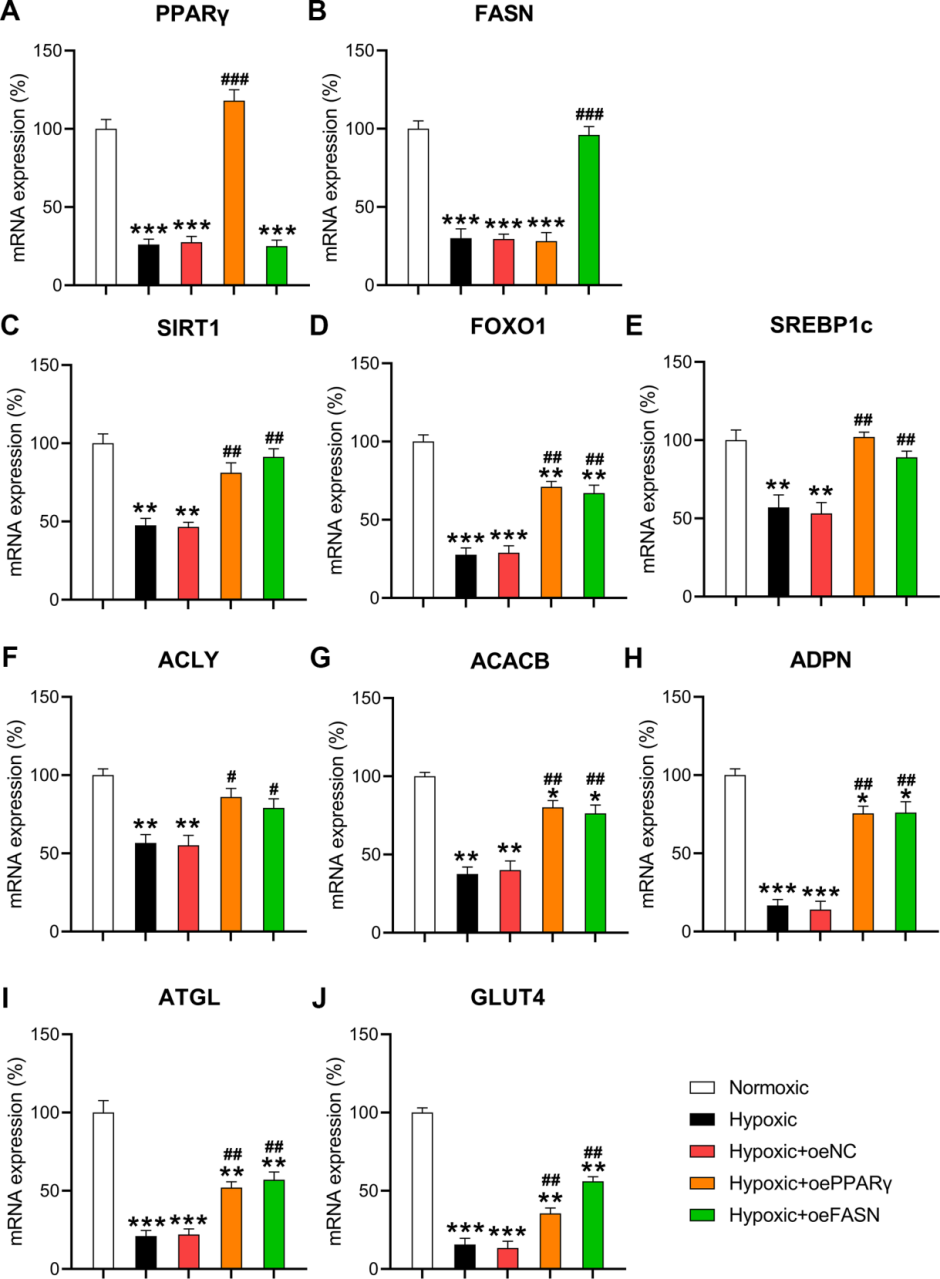
**Effect of PPARγ and FASN overexpression on hypoxia-influenced MR.** Isolated cardiomyocytes were transfected with pcDNA3-NC, pcDNA3-PPARγ, and pcDNA3-FASN for 24 h followed by hypoxia treatment for 24 h. Cells under normoxia served as controls. RT-qPCR was performed to detect the mRNA expression levels of (**A**) PPARγ, (**B**) FASN, (**C**) SIRT1, (**D**) FOXO1, (**E**) SREBP1c, (**F**) ACLY, (**G**) ACACB, (**H**) ADPN, (**I**) ATGL, and (**J**) GLUT4 in the isolated cardiomyocytes in each group (n = 3). *P < 0.05, **P < 0.01, and ***P < 0.001 vs. the normoxia group. ^#^P < 0.05 and ^##^P < 0.01 vs. the hypoxia group.

To validate the role of PPARγ and FASN on the metabolism and death of cardiomyocytes under hypoxic conditions, both proteins were overexpressed in hypoxia-exposed cells. RT-qPCR confirmed that PPARγ and FASN were overexpressed in the hypoxia-exposed cells (Figure 7A, 7B). Then, the expression of metabolic genes, such as SIRT1, FOXO1, SREBP1c, ACLY, ACACB, ADPN, ATGL, and GLUT4, was examined. The results indicated that hypoxia downregulated the expression of these genes. In contrast, the upregulation of PPARγ and FASN partially restored the cellular level of these transcripts in hypoxia-exposed cardiomyocytes (Figure 7C–7J), thereby showing a similar effect to miR-27 and miR-195 inhibition.

**Figure 8:**
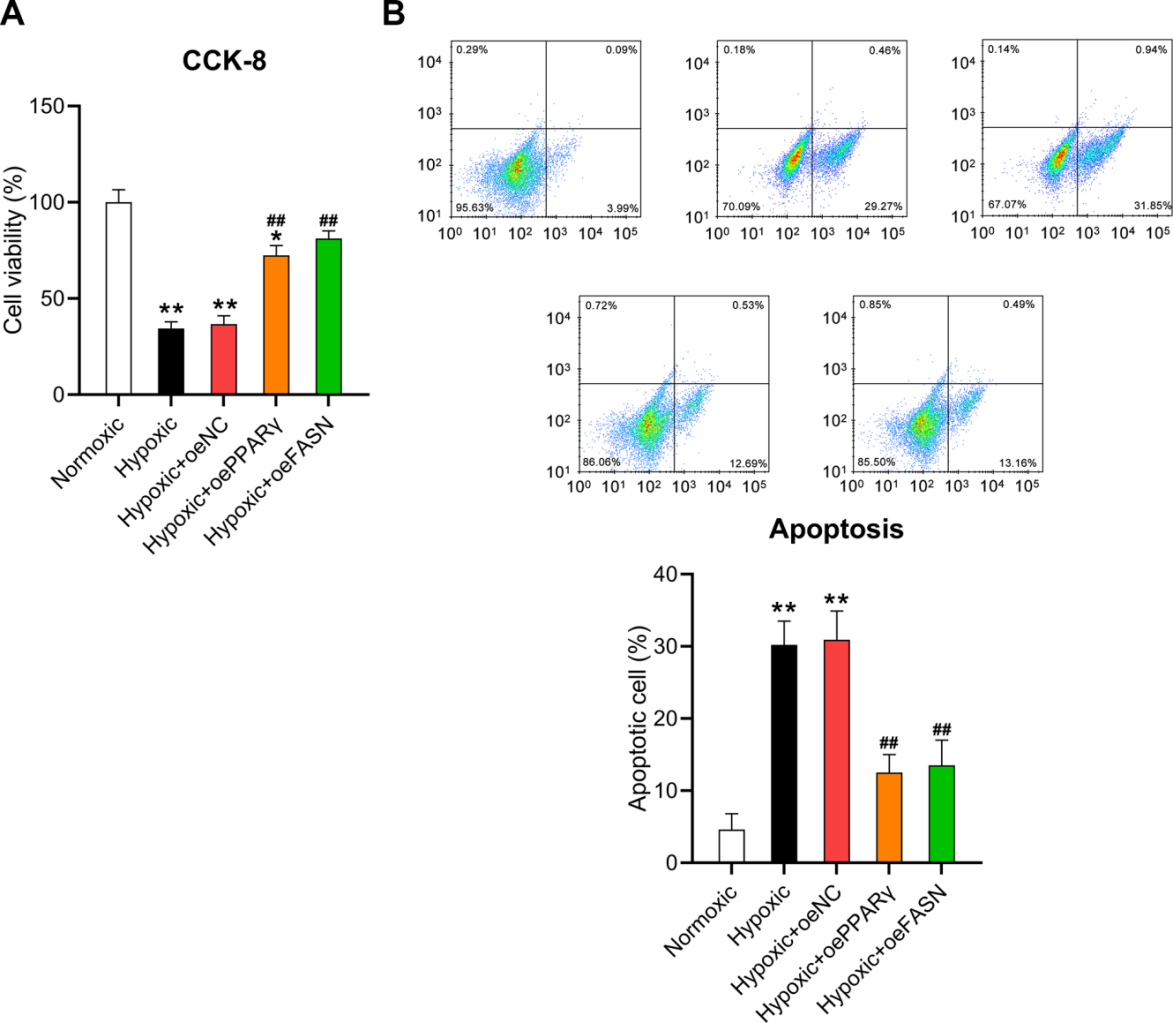
**Effect of PPARγ and FASN overexpression on the viability of hypoxia-induced cardiomyocytes.** Isolated cardiomyocytes were transfected with pcDNA3-NC, pcDNA3-PPARγ, and pcDNA3-FASN for 24 h followed by hypoxia exposure for 24 h. Cells under normoxia served as controls. (**A**) The CCK-8 assay revealed the cell viability in each group. (**B**) Annexin V-FITC/PI flow cytometry was utilized to detect the proportion of apoptotic cardiomyocytes (n = 3). *P < 0.05 and **P < 0.01 vs. the normoxia group. ^##^P < 0.01 vs. the hypoxia group.

Moreover, the CCK-8 assay and flow cytometry were performed to assess the effect of PPARγ and FASN overexpression on the survival and death of cardiomyocytes. The results of the CCK-8 assay revealed that hypoxic conditions significantly inhibited the viability of cardiomyocytes, but overexpressed PPARγ and FASN resulted in recovered cell survival rates (Figure 8A). In addition, flow cytometry revealed that the apoptotic cell proportion in hypoxia-exposed cardiomyocytes was clearly reduced upon overexpression of PPARγ and FASN (Figure 8B), suggesting that PPARγ and FASN are involved in mediating the development of hypoxia-induced cardiomyocyte injury.

## DISCUSSION

Oxygen deficiency (hypoxia) in tissues and cells is the result of an imbalance in oxygen delivery and metabolic requirement that occurs physiologically (e.g., during strenuous exercise or embryonic development) and under pathological conditions (e.g., metabolism-related diseases, ischemia, and cancer). Responses to insufficient oxygen levels include reduced oxygen consumption through metabolic regulation as well as enhanced oxygen delivery by cells via mechanisms, such as increased angiogenesis and erythrocytopoiesis [23]. In the absence of oxygen, cardiomyocytes improve oxygen availability by switching fatty acid oxidation to glycolysis. There are two ways to enable the transfer of metabolic substrates in hypoxic cardiomyocytes. One is the upregulation of HIF-1-induced glucose transporter and glycolytic enzyme expression [24], and the other is the inhibition of PPARγ- and FASN-mediated mitochondrial fatty acid oxidation [25, 26]. Our data indicate that anoxia upregulates HIF-1α and enhances glycolysis while reducing the ATP consumption rate and fatty acid oxidation. We also found that both the PPARγ and FASN levels were reduced under hypoxic conditions. Our data also reveal a potential molecular mechanism for PPARγ and FASN downregulation. The DLRA suggested that miR-27 and miR-195, which were upregulated by hypoxia, targeted the 3’-UTRs of PPARγ and FASN and resulted in downregulation of these two modulators.

Deregulated miRNA expression disrupts the gene regulation system, resulting in MS and related diseases. A previous study reviewed the role of miRNAs in LM [27]. Dyslipidemia is closely related to MS, overweight, glycuresis, and fatty liver disease [28]. miR-122 is involved in the regulation of plasma lipoprotein and intercellular signal molecule levels [29, 30]. miRNAs also affect lipid homeostasis to a significant extent [31]. Key factors involved in LM, such as FABP7 [32], PDCD4 [33], SIRT1 [34], RXRα [35], PPARα [11], ABCG1 [36], and SREBP1 [11, 37], are regulated by miRNAs. PPARs play a pivotal role in the oxidative ability of cellular fatty acids and effectiveness of fatty acid substrates. To cope with short-term hunger, PPARs activate the transcription of fatty acid oxidases [38]. In several anoxic-tolerant organisms (tissue and cells), the FASN pathway serves as a means of balancing redox to meet the need for greater oxidative capacity in conditions of oxygen constraints [39]. In this study, hypoxia was used to disrupt the LM in cardiomyocytes isolated from rats. We found that PPARγ and FASN were significantly downregulated by hypoxia induction, which is consistent with the phenomenon of fatty acid oxidation reduction. Our data also provided evidence that PPARγ and FASN were negatively regulated by miRNAs in hypoxia-induced cardiomyocytes. Many core modules in LM pathways, including SIRT1, FOXO1, SREBP1c, ACLY, ACACB, ADPN, ATGL, and GLUT4, were also upregulated after miRNA inhibition. Our study also explained part of the role of miRNAs in adjusting lipid utilization in hypoxia-induced cardiomyocytes.

Apoptosis during coronary atherosclerosis and ischemia development is a major factor that contributes to cardiomyocyte function failure [40, 41]. In animal models, apoptosis was repeatedly observed in heart tissue under hypoxia treatment [42]. Apoptosis of cardiomyocytes near myocardial infarction areas contribute to the loss of cardiomyocytes, aggravation of cardiac insufficiency, and even HF and death [43]. In addition, obesity is a major risk factor for heart disease, and the dysregulation of LM pathways during hypoxia can be attributed to cardiomyocyte injury. miR-378 was found to adjust the level of caspase-3 in hypoxia-induced cardiomyocyte apoptosis [44]. In the homocysteine-treated endothelial cells of coronary arteries, upregulation of miR-383 reduced the expression of pro-inflammatory cytokines interleukin (IL)-1β, IL-6, and IL-18, leading to inevitable cell death [45]. Here, we observed that hypoxia-associated miR-27 and miR-195 expression was also positively correlated with the level of apoptosis in hypoxia-treated cardiomyocytes. Further validation showed that cell viability was restored by inhibition of miR-27 and miR-195 while apoptosis of hypoxia-treated cardiomyocytes was partially ameliorated by inhibition of miR-27 and miR-195. Additionally, miR-27 and miR-195 were negatively associated with PPARγ and FASN expression and upregulated the LM pathways. These data revealed the role and mechanism of miR-27 and miR-195 in the induced apoptosis of cardiomyocytes under hypoxia.

Our findings show that hypoxia-induced miR-27 and miR-195 are needed to maintain the metabolic state, metabolic transcription pathways, oxidative metabolism, and survival of cardiomyocytes *in vitro*. These results support the idea that anoxia induces cardiac muscle cells to adapt to low oxygen consumption by transforming ATP production from chondriosome aliphatic acid oxidation to glycolysis, demonstrating a molecular role of miRNAs in cardiomyocyte metabolism. This model provides evidence for the functional and metabolic requirements of cardiomyocytes under hypoxia. However, in the unpublished data, we found the ineffective of mimic transfection on metabolism of normoxia condition, rather than hypoxia condition, suggesting that hypoxia-induced miR-27 might only exert its role during hypoxia expose. Further study and investigation will be carried out in future to characterize the role of miRNA in our model. Besides, another limitation of this study is that we did not complete the mechanism by providing experimental explanation for miRNA levels increase during hypoxia.

## MATERIALS AND METHODS

### Cardiomyocyte culture and hypoxia induction

Neonatal rat cardiomyocytes were isolated and cultured as previously described [46]. Briefly, the hearts of neonatal rats (n = 3) were removed aseptically after decapitating the rats. Next, the hearts were minced in serum-free Dulbecco’s Modified Eagle’s Media (DMEM, HyClone, Logan, UT, USA) and digested in 0.25% trypsin. The cell suspension was centrifuged at 2500 rpm for 3 min and the pellet was resuspended in DMEM containing 10% fetal bovine serum. Then, the non-adherent cardiomyocytes were seeded in non-coated culture flasks and cultured in an atmosphere of 5% CO_2_ at 37° C. Hypoxia was induced by placing the cardiomyocytes in a hypoxic chamber (5% CO_2_ and 1% O_2_ at 37° C). The cells cultured in normoxic conditions were maintained in the presence of 20% O_2_. The cardiomyocytes were tested in a series of subsequent tests. Every procedure was approved by the Animal Care and Use Committee of Shenzhen Hospital of Fuwai Hospital.

### miRNA arrays

Cardiomyocytes were seeded in 6-well plates at a density of 10^7^ cells/well. Seventy-two hours after primary isolation, the cells were cultured in either hypoxic or normoxic conditions for an additional 48 h. Total RNA was then extracted from the cells using RNA Stat reagent (Tel-Test, Friendswood, TX, USA). After RNA quality was assessed using the Agilent bioanalyzer system (Santa Clara, CA, USA), the samples were shipped to Exiqon (Vedbaek, Denmark), where hybridizations were performed in-house. The following two-color hybridizations using cyanine 3 (Cy3) and Cy5 labeling were performed: hypoxia vs. normoxia (n = 3 independent biological samples in each group). To minimize the effects of dye bias, the raw microarray data were then normalized in-house by Exiqon using M vs. A plots, in which the log_2_ intensity ratio between the two labeled samples is plotted against the log_2_ mean intensity of the two labeled samples. The locally weighted scatterplot smoothing regression algorithm was then applied to the data to generate normalized log_2_ Cy3-to-Cy5 ratios [47]. To generate uncorrected P values for the fold change data from the microarray, one-sample *t*-testing was performed. Correction for multiple comparisons was performed using the Bonferroni step-down method [48]. All microarray data were submitted to the Gene Expression Omnibus at the National Center for Biotechnology Information (accession no. GSE24954; Bethesda, MD, USA).

### Cell transfections

The mimics (10 nM) and inhibitors (100 nM) of miR-27 and miR-195 as well as the corresponding negative control (NC) (GenePharma, Shanghai, China) were transfected into hypoxia-induced cardiomyocytes. Transfection of mimics or inhibitors was conducted using Lipofectamine 2000 reagent (Invitrogen, Carlsbad, CA, USA).

### Real-time quantitative PCR (RT-qPCR)

TRIzol reagent was used to isolate total RNA from the heart tissue, which was purified using an RNeasy kit (Qiagen, Hilden, Germany). After reverse transcription (SuperScript III; Invitrogen), TaqMan probes (Applied Biosystems, Foster City, CA, USA) were used to quantify microRNA expression and SYBR Green for quantifying mRNA expression using quantitative RT-qPCR on the StepOnePlus thermocycler (Applied Biosystems). PerfeCTa ToughMix and PerfeCTa SYBR Green SuperMix (Quantabio, Beverly, MA, USA) were used for performing TaqMan and SYBR Green RT-qPCR, respectively. The 2^−ΔΔCT^ method was used to analyze the changes in related gene expression and drew with standardized relative units. Ubiquitin C (UBC), beta-2-microglobulin (β2M), glyceraldehyde 3-phosphate dehydrogenase (GAPDH), and 18S ribosomal RNA (rRNA) were used as the internal controls, as the expression of these molecules remained constant in cardiomyocytes cultured in hypoxic conditions. The following primer sequences were used: HIF-1α, 5’-TGA TGT GGG TGC TGG TGT C-3’ (forward) and 5’-TTG TGT TGG GGC AGT ACT G-3’ (reverse); muscle carnitine palmitoyltransferase I (mCPT-I), 5’-GGA GAG TGC CAG GAG GTC ATA G-3’ (forward) and 5’-TGT CCT TTG TAA TGT GCG AGC TG-3’ (reverse); vascular endothelial growth factor (VEGF), 5’- CGG AAG ATT AGG GAG TTT-3’ (forward) and 5’-GGA TGG GTT TGT CGT GTT-3’ (reverse); SIRT1, 5’-CAA CTT GTA CGA CGA AGA C-3’ (forward) and 5’-TCA TCA CCG AAC AGA AGG-3’ (reverse); forkhead box protein O1 (FOXO1), 5’-TCC TCG AAC CAG CTC AAA CG-3’ (forward) and 5’-GGC GGT GCA AAT GAA TAG CAA G-3’ (reverse); SREBP1c, 5’-GGA GCC ATG GAT TGC ACA TT-3’ (forward) and 5’-AGG AAG GCT TCC AGA GAG GA-3’ (reverse); ATP citrate lyase (ACLY), 5’-TCT GGG AGG TGT CAA CGA G-3’ (forward) and 5’-GGT CTT GGC ATA GTC ATA GGT-3’ (reverse); acetyl-coenzyme A (CoA) carboxylase (ACACB), 5’-ACT ATG AGG CCC AGC ATG TC-3’ (forward) and 5’-TGA CCC TAT TGC CTC CAA AG-3’ (reverse); adiponutrin (ADPN), 5’-GAT GGA GGA GTG AGT GAC AA-3’ (forward) and 5’-CTG AAT GCA TCC AAA TAT CC-3’ (reverse); adipose triglyceride lipase (ATGL), 5’-TCA CGG TAC CGG ATC CTT CCC GAG GGA GAC CAA GTG G-3’ (forward) and 5’-TCA AAG AGC GTA ATC TGG AAC ATC GTA TGG-3’ (reverse); glucose transporter type 4 (GLUT4), 5’-CCA ACT GGA CCG CCA ACT T-3’ (forward) and 5’-GAG CCC GGC GAA GAT CA-3’ (reverse); PPARγ, 5’-TCG CTG ATG CAC TGC CTA TG-3’ (forward) and 5’-GAG AGG TCC ACA GAG CTG ATT-3’ (reverse); FASN, 5’-GGA GGT GGT GAT AGC CGG TAT-3’ (forward) and 5’-TGG GTA ATC CAT AGA GCC CAG-3’ (reverse); 18S rRNA, 5’-CTA CCA CAT CCA AGG AAG CA-3’ (forward) and 5’-TTT TTC GTC ACT ACC TCC CCG-3’ (reverse); β2M, 5’-GAA GCG ATT CTA GGG AGC AG-3’ (forward) and 5’-GGA GCA GCA TTC TGA GTA GA-3’ (reverse); UBC, 5’-GTG TCT AAG TTT CCC CTT TTA AGG-3’ (forward) and 5’-TTG GGA ATG CAA CAA CTT TAT TG-3’ (reverse); GAPDH, 5’-GAA GGT GAA GGT CGG AGT C-3’ (forward) and 5’-GAA GAT GGT GAT GGG ATT TC-3’ (reverse); U6, 5’-CTC GCT TCG GCA GCA CA-3’ (forward) and 5’-AAC GCT TCA CGA ATT TGC GT-3’ (reverse).

### Western blotting (WB)

Whole cell lysate was prepared by lysing cultured cells in radioimmunoprecipitation assay buffer (pH 8.0) containing protease inhibitor cocktail (Roche Applied Science, Basel, Switzerland). Protein concentrations were determined using a bicinchoninic acid (BCA) kit. Proteins were separated using sodium dodecyl sulfate polyacrylamide gel electrophoresis and transferred onto a polyvinylidene difluoride membrane (Millipore, Burlington, MA, USA). The membrane was probed overnight with primary antibodies at 4° C and washed with tris-buffered saline-Tween 20 (TBST). The washed membrane was probed with secondary antibodies for 1 h at room temperature. The following antibodies were used: FASN (ab99359; 270 kDa; 1:500; Abcam, Cambridge, UK), PPAR-γ (ab59256; 54 kDa; 1:100; Abcam), Bax (ab53154; 25 kDa; 1:1000; Abcam), cleaved caspase-9 (ab2324; 40 kDa; 1:1000; Abcam), caspase-9 (ab25758; 50 kDa; 1:1000; Abcam), and Actin (ab8227; 43 kDa; 1:1000; Abcam). After several washes with TBST, the bands on the membrane were detected using the SuperSignal West Femto Maximum Sensitivity Substrate kit (Thermo Fisher Scientific, Waltham, MA, USA).

### ATP consumption, glycolysis, and oxidation measurement

ATP levels in cardiomyocytes were assessed using standardized high-performance liquid chromatography methods [49]. Glycolysis as well as oxidation of palmitate, glucose, and lactate were determined by the quantitative measurement of ^3^H_2_O or ^14^CO_2_ produced by cardiomyocytes perfused with either KH solution containing [9,10-^3^H]palmitate and [U-^14^C]lactate or [U-^14^C]glucose and 5-[^3^H]glucose as described in a previous report [50].

### 5-Ethynyl-2’-deoxyuridine (EdU) staining

Cardiomyocytes were washed three times with phosphate-buffered saline (PBS) and stained with 300 μL of EdU. After three washes with PBS, cardiomyocytes were observed under a fluorescence microscope (Olympus, Tokyo, Japan). Red staining indicated proliferating cardiomyocytes, and the number of multiplying cardiomyocytes in six different fields was counted.

### Cell counting kit-8 (CCK-8) assay

The CCK-8 assay was used to evaluate cell proliferation. Cardiomyocytes were cultured in 96-well plate; then CCK-8 solution (10 μL) was added in every well, and the cardiomyocytes were incubated with the solution for 2 h at 37° C. Optical density at 450 nm was measured using an automatic microplate reader (Infinite M200; Tecan, Männedorf, Switzerland).

### Flow cytometry

Cardiomyocyte apoptosis was evaluated using the FITC Annexin V Apoptosis Detection kit with propidium iodide (PI) (BD Pharmingen, San Diego, CA, USA). The cardiomyocytes were cultured in 20 μL binding buffer; PI (5 μL) and annexin V-FITC (10 μL) were added to the cardiomyocyte suspension. The cells were then incubated at room temperature in the dark for 30 min. Flow cytometric analysis was performed using a FACScan device (Beckman Coulter, Fullerton, CA, USA). The data were analyzed using FlowJo software (BD Biosciences, San Jose, CA, USA).

### Dual-luciferase reporter assay (DLRA)

In order to investigate whether miR-27 and miR-195 targeted PPARγ and FASN, wild type (WT) and mutant (MU) 3’-untranslated regions (3’-UTRs) of PPARγ and FASN were analyzed using the DLRA. Luminescence was calibrated based on the firefly luciferase sequence, and Renilla luciferase was used as the reporter luminescent. Cardiomyocytes were transfected with miR-27 and miR-195 mimics, NC mimics, and luciferase vectors for 36 h.

### TargetScan prediction

miR-27 and miR-195 were identified using the TargetScan and miRDip [51] prediction algorithm. On the website (http://www.targetscan.org and http://ophid.utoronto.ca/mirDIP), the predictions were ranked according to the predicted efficacy of targeting [52].

### Statistical analysis

The data are presented as means ± standard deviations. A one-way analysis of variance was used to compare the different groups, and *t*-tests were used for comparisons between two groups. A P value < 0.05 indicated significant differences.
